# Differential Effects of Social Vulnerability Index Among Older and Younger Adults With Cancer

**DOI:** 10.1093/geroni/igaf122.1209

**Published:** 2025-12-31

**Authors:** Mackenzie Fowler, Geetanjali Saini, Elizabeth Baker, Mahak Bhargava, Ritu Aneja

**Affiliations:** The University of Alabama at Birmingham, Birmingham, Alabama, United States; The University of Alabama at Birmingham, Birmingham, Alabama, United States; The University of Alabama at Birmingham, Birmingham, Alabama, United States; The University of Alabama at Birmingham, Birmingham, Alabama, United States; The University of Alabama at Birmingham, Birmingham, Alabama, United States

## Abstract

Social determinants of health, (e.g. social vulnerability index [SVI]), are associated with adverse cancer-related outcomes. Most cancer cases occur among older adults (≥65). Effects of SDOH may differ between older and younger adults. Our objective was to examine the role of SVI on all-cause mortality among adults with cancer in Alabama. We included adults (≥18) with incident breast, prostate, colorectal, or lung cancer between 1/1/2010 and 12/31/2019 from the Statewide Cancer Registry. The exposure was census tract-level SVI in quartiles. The outcome was overall survival (OS) from diagnosis to death or end of follow-up (12/31/2021). We performed Cox proportional hazards models to estimate associations adjusting for age, race, sex (if applicable), stage, census-tract rurality (Rural-Urban Commuting Area Code), and subtype (breast). We assessed interaction and stratified by age group at diagnosis (<65, ≥65). We further adjusted for insurance status. Living in higher SVI quartiles was associated with significantly higher hazard of mortality across all cancer types examined; this association was stronger for those <65 at diagnosis as interaction with age was significant for breast, prostate, and colorectal cancer (p-values: breast: 0.025; prostate: <0.001; colorectal: 0.031). Adjusting for insurance status, the association between SVI remained significant among older adults but not among younger adults. Insurance status had a stronger independent association with mortality among younger adults. These results indicate neighborhood-level SVI and individual-level insurance status may have greater negative effect on OS among younger adults whereas other unmeasured factors may affect OS in cancer among older adults.

